# SOCIAL ROLES AND PERSONALITY IN LATER LIFE

**DOI:** 10.1093/geroni/igz038.2673

**Published:** 2019-11-08

**Authors:** Anna E Kornadt, Anna E Kornadt

**Affiliations:** 1 Bielefeld University, Bielefeld, Germany; 2 Bielefeld University, Bielefeld, Nordrhein-Westfalen, Germany

## Abstract

Despite considerable stability of the Big Five personality traits, there is evidence for personality plasticity and change across the lifespan. In younger years, the investment in social roles, such as entering worklife or starting a family has been shown to drive personality change. With regard to personality in later life, the investigation of social roles has so far been neglected. A questionnaire was developed to assess a large number of social roles that can be assumed in the second half of life. N = 306 participants aged 50 to 86 years reported on their social roles and rated their personality traits. Results show that assuming and investing in certain social roles (e.g. friend, retiree, volunteer) mediated the effects of age on the Big Five, especially for the oldest participants and in the domains openness and extraversion. The findings support the importance of social roles for personality also in later life.

